# Coexistence of Brachial Plexus-Anterior Scalene and Sciatic Nerve-Piriformis Variants

**DOI:** 10.7759/cureus.9115

**Published:** 2020-07-10

**Authors:** Jean-Marc P Lucas, Ali Sandouka, Oren D Rosenthal

**Affiliations:** 1 Osteopathic Medicine, Lake Erie College of Osteopathic Medicine, Bradenton, USA; 2 Anatomy, Lake Erie College of Osteopathic Medicine, Bradenton, USA

**Keywords:** scalene muscles, anterior scalene variation, anatomic variation, brachial plexus, thoracic outlet syndrome, sciatic nerve, piriformis variation, piriformis syndrome

## Abstract

The trunks of the brachial plexus typically pass through the interscalene triangle, between the anterior and middle scalene muscles and superior to the first rib. Likewise, the two components of the sciatic nerve, tibial and common fibular nerves, usually join and pass together inferior to the piriformis muscle. We present a cadaver with anatomic variations of both the right brachial plexus-interscalene triangle relationship and the sciatic nerve-piriformis relationship. The right brachial plexus C5 and C6 roots formed the superior trunk as they passed through a bifurcated anterior scalene muscle, while the C7, C8, and T1 roots passed posterior to the anterior scalene. After passing through the left greater sciatic foramen, the sciatic nerve branched into the common fibular and tibial nerves, which passed through and inferior to the piriformis muscle, respectively. The presence of these anatomic variations may predispose individuals to symptomatic nerve entrapments such as thoracic outlet syndrome and piriformis syndrome. This finding is relevant to clinicians performing invasive procedures and diagnosing neurological conditions.

## Introduction

The brachial plexus (roots C5-T1) is a group of nerves that supply sensory and motor function to the upper extremity [1]. The roots of the brachial plexus and the subclavian vessels are classically described as residing within the interscalene triangle. The interscalene triangle is a space between the anterior and middle scalene muscles and first rib, forming the anterior, posterior, and inferior borders of the triangle, respectively. The sciatic nerve (roots L4-S3) is classically described as traveling inferior to the piriformis muscle and branches into the common fibular (peroneal) (roots L4-S2) and tibial (roots L4-S3) nerves in the popliteal fossa [2-5]. The common fibular nerve supplies sensory and motor innervation to the anterior and lateral leg [4]. The tibial nerve supplies sensory and motor innervation to the posterior thigh, leg, and plantar surface of the foot [5]. Variations in brachial plexus and sciatic nerve location are relatively common. The superior trunk of the brachial plexus has been reported to pierce the anterior scalene muscle between 6.5% and 15% of people, whereas the sciatic nerve may send its common fibular branch through the piriformis in up to 12% of people [6-8]. When present, an accessory scalene muscle (scalenus accessorious) may originate superiorly from the anterior or medial surface of the middle scalene and insert into the caudal anterior scalene tendon [9,10]. A smaller scalene muscle fascicle, scalenus minimus, is reported to usually arise from the transverse process of C6 or C7 and insert posterior to the subclavian artery into both the first rib and suprapleural membrane (Sibson’s fascia) of the lung cupula [11]. The relationship between brachial plexus and sciatic nerve variations has not been previously established. In this study, we present a case of one cadaver with copresenting variations of the right brachial plexus-anterior scalene muscle relationship and left sciatic nerve-piriformis muscle relationship. 

## Case presentation

**Figure 1:**
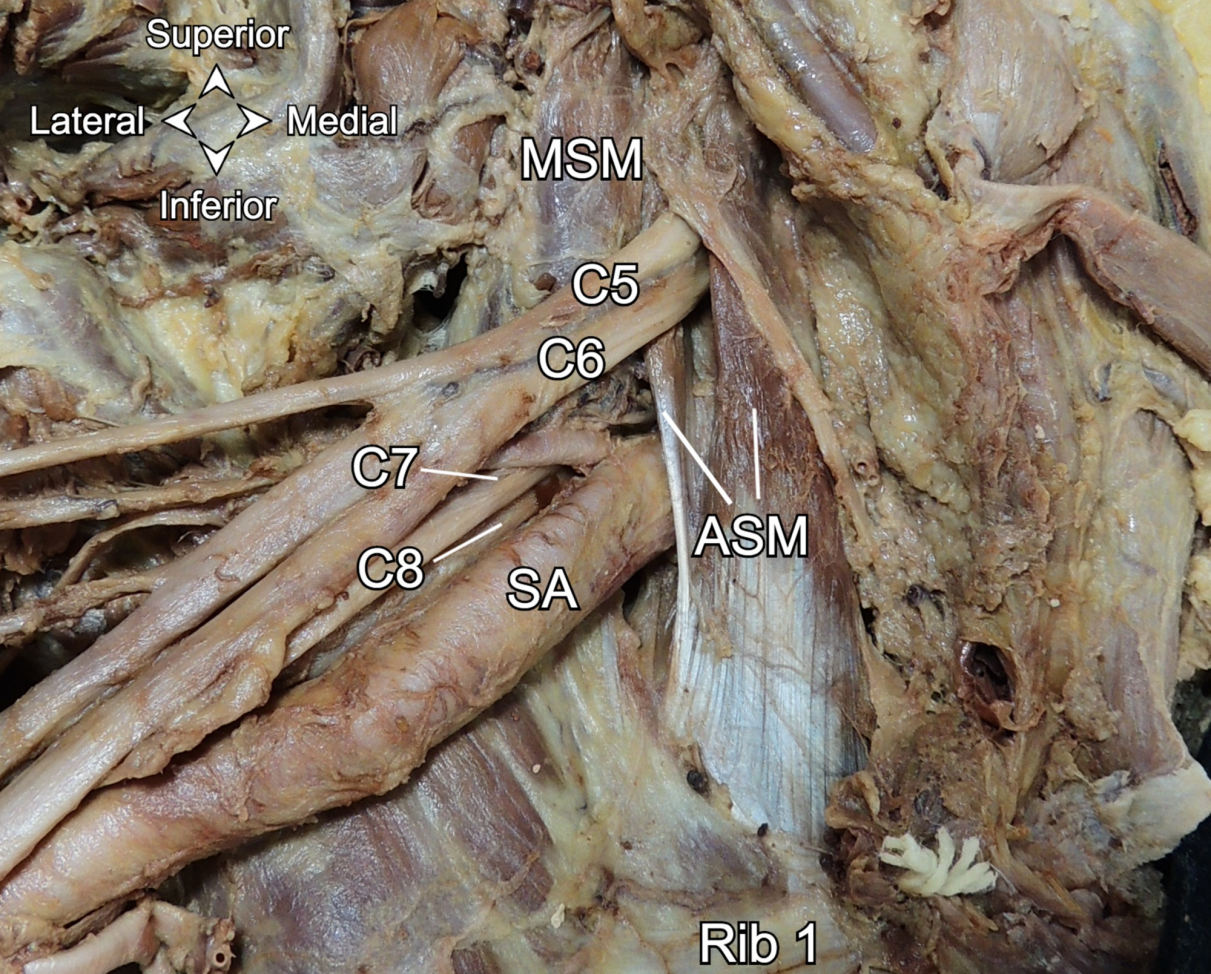
Anterior view of the right C5 and C6 roots passing through the anterior scalene muscle. ASM: anterior scalene muscle. MSM: middle scalene muscle. SA: subclavian artery.

During routine cadaveric dissection of a 72-year-old female in the Gross Anatomy Laboratory at Lake Erie College of Osteopathic Medicine Bradenton campus, a right brachial plexus and a left sciatic nerve variation were noted. The neck was dissected bilaterally to expose the scalene muscles and the brachial plexus. It was noted that the right C5 and C6 brachial plexus roots passed as the superior trunk through the anterior scalene muscle (Figure 1).

**Figure 2:**
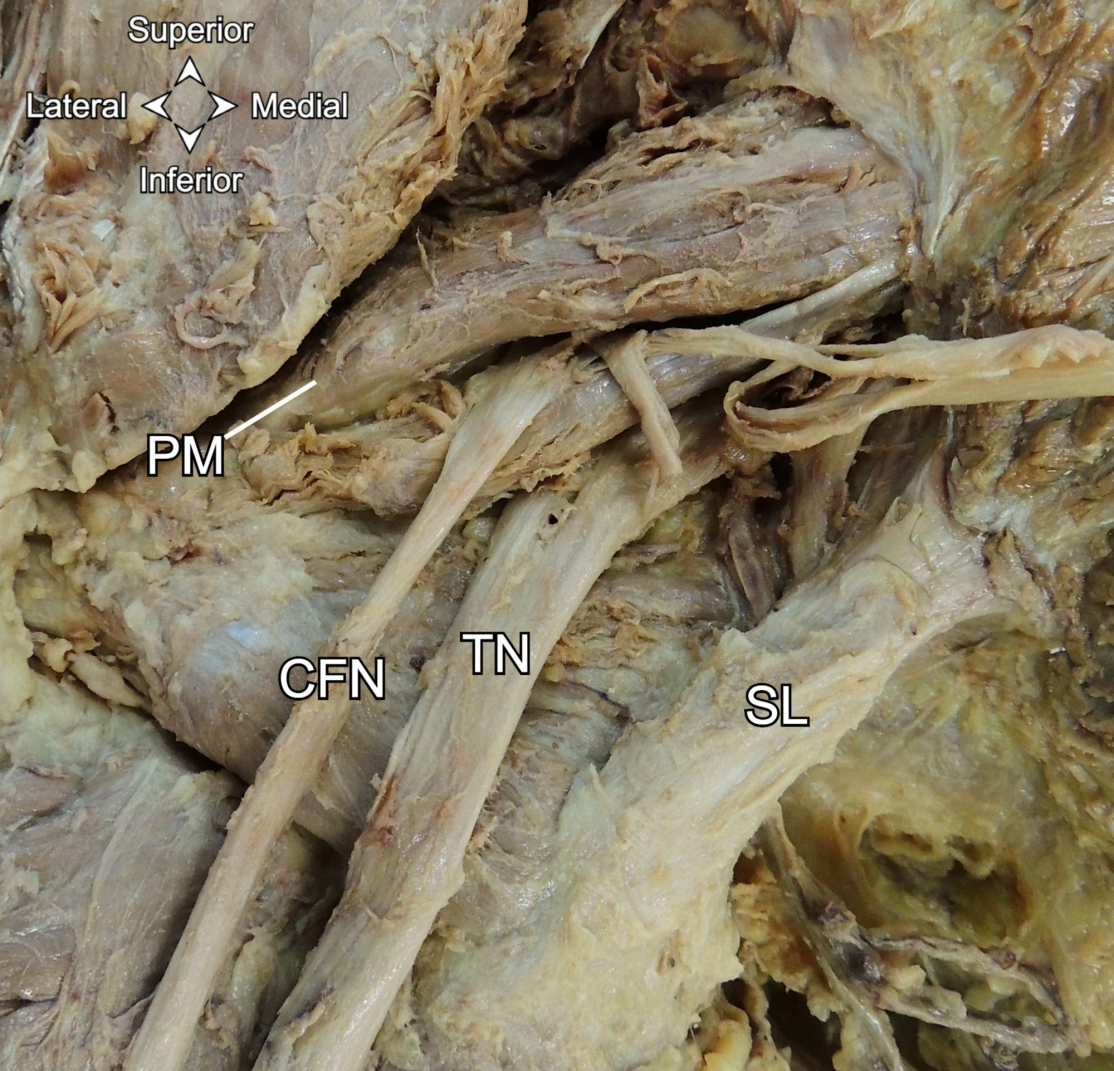
Posterior view of the divided left sciatic nerve. PM: piriformis muscle. CFN: common fibular nerve. TN: tibial nerve. SL: sacrotuberous ligament.

The left C5 and C6 brachial plexus roots passed posterior to the anterior scalene. The superficial gluteal muscles were reflected bilaterally, exposing the piriformis muscles and sciatic nerves. The left sciatic nerve passed through the left greater sciatic foramen and immediately branched into the common fibular and tibial nerves. The common fibular nerve then pierced the left piriformis, while the tibial nerve passed inferior to the left piriformis (Figure 2). The left common fibular and tibial nerves rejoined to form the sciatic nerve inferior to the inferior edge of the piriformis. The left sciatic nerve then branched into the common fibular and tibial nerves again upon entering the popliteal fossa. The right sciatic nerve passed undivided under the right piriformis muscle and divided near the popliteal fossa. 

## Discussion

Neuromuscular variations of the brachial plexus and sciatic nerve are well documented independently of each other. No cases have been described in the literature reporting the presence of contralateral brachial plexus trunk and sciatic nerve variations in the same cadaver. The present right brachial plexus-anterior scalene anomaly represents a type 2 variation according to the Keet and Louw classification system [12]. Likewise, the present sciatic nerve anomaly represents a type 2 variation, according to the Beaton and Anson classification system [13]. It is possible other investigators have missed this copresentation because they were not exploring both anatomic regions. Knowledge of such a correlation may have clinical ramifications on neurological diagnosis and local invasive procedures. 

The presence of these two anatomic variations may predispose individuals to pain and paresthesias, such as those experienced with thoracic outlet syndrome (TOS) and to a lesser extent, piriformis syndrome (PS). An individual with both variations, such as this case, could present with both upper and lower limb neurological symptoms. TOS is due to the compression of some or all parts of the brachial plexus with or without subclavian vessel compression from the neck to the axilla [7]. Leonhard et al. demonstrated that 50% of subjects with brachial plexus variants that pierce the anterior scalene display TOS symptoms, whereas 13.9% of subjects with classic (type 1) variations were symptomatic [14]. Patients with piercing variants are thus more likely to experience pain, paresthesias, and weakness along the dermatomes and myotomes supplied by the roots piercing the anterior scalene [7]. However, people with sciatic nerve variations are equally as likely (16.2%) as people with classic (type 1) anatomy to have PS [3]. This discrepancy between TOS and PS anatomic etiology may warrant further investigation. Additionally, Leonhard et al. suggests that provocative tests yield false negative results for about half of all cases of TOS due to anatomical variation [14]. Therefore, anatomic variation may account for more cases of TOS than what is reported in the literature. When examining and treating a patient with TOS signs and symptoms, ultrasound is a reliable means of ruling out anatomic variation. Ultrasound should also be used to evaluate patients undergoing deep gluteal procedures due to the relatively high prevalence of sciatic nerve variation. 

The anterior scalene in this case appears bifurcated with the C5 and C6 roots passing between the two muscle bellies. Each anterior scalene belly becomes tendinous prior to their joining at the first rib. Paraskevas et al. reported an accessory scalene muscle impinging on the middle and lower trunks of the brachial plexus [9]. In contrast, the scalene variant in our case appears to contact only the superior trunk. Additionally, it does not appear to arise from the middle scalene proximally as is described for an accessory scalene. While we do not have the medical history from our cadaver, nerve entrapment is plausible and may have been a source of neurological symptoms along the C5 and C6 and L4-S2 dermatomes and myotomes for our cadaver in life. Anatomic sources of TOS and PS may be exacerbated by hypertrophy due to repetitive shoulder and hip activities, atrophy from underuse, or abnormal changes in muscle length due to sustained poor posture [15,16].

## Conclusions

The brachial plexus has been described as traveling through the interscalene triangle. The sciatic nerve most commonly travels with its two component nerves conjoined and passing inferior to the piriformis muscle. Anatomic variation is relatively commonplace in these two regions, but copresenting variation in both areas has not been reported until now. This finding may have implications when patients report to clinicians with peripheral nerve entrapment signs and symptoms. Knowledge of these two variations, both individually and in this copresentation, is clinically relevant for invasive procedures and diagnosis of neurological conditions. This would be especially relevant if a patient presented with simultaneous upper and lower limb neurological impairments. 
